# IL-6 Deficiency Attenuates Skeletal Muscle Atrophy by Inhibiting Mitochondrial ROS Production through the Upregulation of PGC-1*α* in Septic Mice

**DOI:** 10.1155/2022/9148246

**Published:** 2022-04-27

**Authors:** Bo Yang, Xiaoming Yang, Xiangran Sun, Jiaofang Shi, Yi Shen, Ren Chen

**Affiliations:** ^1^Department of Orthopedics, Renmin Hospital of Wuhan University, Wuhan, Hubei 4300670, China; ^2^Department of Orthopedics, Renmin Hospital of Yangxin Country, Huangshi, Hubei 435200, China; ^3^Department of Orthopedics, Traditional Chinese Medicine Hospital of Guangshui, Guangshui, Hubei 432700, China

## Abstract

Current evidences indicate that both inflammation and oxidative stress contribute to the pathogenesis of sepsis-associated skeletal muscle atrophy. However, the interaction between inflammation and oxidative stress has not been completely understood in sepsis-associated skeletal muscle atrophy. Here in the present study, a murine model of sepsis has been established by cecal ligation and puncture (CLP) with wild-type and interleukin- (IL-) 6 knockout (KO) mice. Our results suggested that IL-6 KO largely attenuated skeletal muscle atrophy as reflected by reduced protein degradation, increased cross-sectional area (CSA) of myofibers, and improved muscle contractile function (all *P* < 0.05). In addition, we observed that IL-6 KO promoted the expression of peroxisome proliferator-activated receptor *γ* coactivator–1alpha (PGC–1*α*) and inhibited CLP-induced mitochondrial reactive oxygen species (ROS) production in skeletal muscles (all *P* < 0.05). However, the knockdown of PGC–1*α* abolished the protective effects of IL-6 KO in CLP-induced skeletal muscle atrophy and reversed the changes in mitochondrial ROS production (all *P* < 0.05). Ex vivo experiments found that exogenous IL-6 inhibited PGC–1*α* expression, promoted mitochondrial ROS production, and induced proteolysis in C2C12 cells (all *P* < 0.05). Together, these results suggested that IL-6 deficiency attenuated skeletal muscle atrophy by inhibiting mitochondrial ROS production through the upregulation of PGC–1*α* expression in septic mice.

## 1. Introduction

Sepsis is defined as an uncontrolled systemic inflammatory response associated with severe infection. It strongly affects patients' clinical outcomes due to accompanied multiple organ injuries [1]. Skeletal muscle atrophy is a common complication among septic patients admitted to the intensive care units (ICU) [2]. The molecular mechanism which underpins skeletal muscle atrophy following sepsis is complex. Accumulating evidence suggested that sepsis-induced continued protein loss of myofibers finally results in decreased muscle force-generating capacity and muscle fiber atrophy [3, 4]. The overexpression of proinflammatory cytokine IL-6 has previously been reported to cause muscle atrophy through the downregulation of the phosphorylation of ribosomal S6 kinase and the induction of muscle degradation via JAK/STAT, FOXO3a, and Atrogin-1 upregulation [5, 6]. In addition, it has been widely reported that the inhibition of IL-6 signaling can largely attenuate skeletal muscle atrophy by reducing protein degradation [7, 8]. However, the underlying mechanism by which IL-6 promotes protein degradation in skeletal muscle has not been completely understood.

Skeletal muscle atrophy results from the imbalance between protein synthesis and degradation. Current evidence suggested that protein degradation appears to be dominant in developing muscle atrophy during sepsis [9]. Protein degradation within skeletal muscle is associated with three major proteolysis-related signaling pathways: ubiquitin-proteasome system (UPS), autophagy, and calpains [10]. The muscle atrophy gene-1 (Atrogin-1) and muscle RING finger 1 (MuRF-1) are two key positive regulators of UPS-mediated proteolysis, and sepsis largely upregulates the expression of Atrogin-1 and MuRF in skeletal muscles [11]. The production of reactive oxygen species (ROS), especially mitochondrial ROS, has been believed to be essential for the activation of UPS and subsequent protein degradation in numerous types of muscle atrophy [12–14]. It has also been reported that the overproduction of ROS following sepsis contributes to skeletal muscle atrophy [15]. The peroxisome proliferator-activated receptor *γ* coactivator–1alpha (PGC–1*α*), a master regulator of mitochondrial biogenesis, deficiency has been believed to be a major cause of oxidative stress and skeletal muscle atrophy [16, 17]. It has been demonstrated that IL-6 could suppress PGC–1*α* expression [18] and promote mitochondrial ROS production [19]. However, whether the protective effects of IL-6 inhibition are associated with the expression levels of PGC–1*α* and ROS production remains uncertain.

The present study was designed to test the hypothesis that IL-6 induces skeletal muscle atrophy by promoting mitochondrial ROS production through the downregulation of PGC–1*α* expression in septic mice. Wild-type and IL-6 knockout (KO) mice were used to establish a murine model of sepsis by cecal ligation puncture (CLP). Our results found that IL-6 KO largely attenuated diaphragm dysfunction by inhibiting mitochondrial ROS production through the upregulation of PGC-1ɑ in septic mice. In addition, we also determined whether the inhibition of IL-6 trans-signaling with sgp130Fc can protect septic mice against skeletal muscle atrophy. Our results suggested that sgp130Fc promoted PGC-1ɑ expression, downregulated mitochondrial ROS production, and reduced protein degradation. Finally, sgp130Fc markedly alleviated diaphragm atrophy and contractile dysfunction.

## 2. Methods and Materials

### 2.1. Study Design and Grouping

To investigate the roles of IL-6 KO in sepsis-induced skeletal muscle atrophy. Wild-type and IL-6 KO mice were randomly assigned into the following groups: (1) a wild-type Sham group (WT-Sham, *n* = 6): wild-type C57BL/6 mice (IL-6^+/+^) were sham-operated (laparotomy without CLP); (2) a wild-type CLP group (WT-CLP, *n* = 6): wild-type C57BL/6 mice (IL-6^+/+^) received CLP; (3) an IL-6 KO Sham group (IL-6 KO-Sham, n =6): IL-6 KO mice (IL-6^−/−^) were sham-operated; (4) an IL-6 KO CLP group (IL-6 KO-CLP, *n* = 6): IL-6 KO mice (IL-6^−/−^) received CLP.

Animals were kept for 48 hours after sham operation or CLP. Then, animals were killed under anesthesia by cervical dislocation. Blood samples were collected for blood gases analysis and the measurements of serum lactate levels. The extensor digitorium longus (EDL) muscles were collected to detect of protein degradation, the mitochondrial ROS production, the immunofluorescence staining for the measurements of cross-sectional area (CSA) myofibers, and the measurements of muscle force-generating capacity. Then, the experiment was conducted to determine whether IL-6 KO protects septic mice against skeletal muscle atrophy through the upregulation of PGC-1ɑ. Animals were assigned to the following groups: (1) a wild-type CLP group (WT-CLP, *n* = 6): wild-type C57BL/6 mice (IL-6^+/+^) received CLP; (2) an IL-6 KO CLP group (IL-6 KO-CLP, *n* = 6): IL-6 KO mice (IL-6^−/−^) received CLP; (3) an IL − 6 KO CLP + shCTRL group (IL − 6 KO − CLP + shCTRL): IL-6 KO mice (IL-6^−/−^) received CLP and transfection of negative control shRNA; and (4) an IL − 6 KO CLP + shPGC − 1*ɑ* group (IL − 6 KO − CLP + shPGC − 1*ɑ*): IL-6 KO mice (IL-6^−/−^) received CLP and transfection of PGC-1ɑ shRNA. Negative control siRNA and PGC-1ɑ shRNA were intubated with in vivo fectamine (IVF3005; Thermo Fisher Scientific, MA, USA) and injected through the tail veins. RT-qPCR and western blots were performed to determine the transfection efficiency. Animals were kept for 48 hours after CLP. At the end of experiments, blood and the extensor digitorium longus (EDL) muscles were collected for biochemical and histological analysis as described above.

### 2.2. Animal Preparation

Eight-week-old wild-type male C57BL/6 mice were purchased from Hubei Provincial Center for Disease Control and Prevention (Wuhan, China). IL-6 knockout (KO) mice (IL-6^−/−^) were purchased from Cyagen Biosciences (Hangzhou, China). As previously described, we used IL-6 KO mice of the C57BL/6 background [20]. Mice were kept in separate cages under controlled conditions with a 12 : 12 light-dark cycle and fed ad libitum for one week of weight control. All procedures were performed following the guidelines of Animal Care and Use. This study was approved by the institutional animal experiment ethics committee (AUP: SQ20200127).

### 2.3. Murine Model of Sepsis

A murine model of sepsis was established by cecal ligation puncture (CLP) as previously described [21]. In brief, laparotomy (1-cm incision) was performed under anesthesia (pentobarbital, 50 mg/kg, i.p.) and skin preparation and sterilization. The cecum was ligated at the middle of the cecum and punctured twice. Then, the incision was closed and sterilized. The sham-operated mice received laparotomy, and the cecum was manipulated without ligation and puncture. The murine model of sepsis was considered to be successfully established if fever (increased rectal temperature > 1°C), increased heart rate (2 times higher than normal control), and respiration rate (2 times higher than normal control) were observed. In addition, the serum levels of lactate and the mean arterial blood pressures were measured.

### 2.4. Cell Culture and Plasmid Preparation

C2C12 murine myoblasts were purchased from American Type Culture Collection (Manassas, VA, USA) and cultured at 37°C in 5% CO_2_ and 95% atmospheric air. Cells were maintained in DMEM supplemented with 10% fetal bovine serum and antibiotics. Cells were transfected with adenovirus of interest and harvested 48 hours after infection for biological measurements. The shPGC-1*α* target sequence is “GCAACATGCTCAAGCCAAACC”. The control target sequence is “GTTCTCCGAACGTGTCACGT”. pcDNA3.1-PGC-1*α* for the expression of human full-length PGC-1*α* was synthesized by Biofavor (Wuhan, China). Adenoviral vectors expressing PGC-1*α* were generated using the AdEasy system.

### 2.5. Blood Analysis

At the end of the experiments, blood samples were collected in tubes with EDTA for anticoagulation. Then, blood gas analysis and the measurements of serum lactate were performed. Arterial pH, PaO_2_, PaCO_2_, HCO_3_^–^, and lactate were measured using a portable device (i–STAT1Analyzer, Abbott, Kyoto, Japan).

### 2.6. Immunofluorescence Staining of Myofibers

Immunofluorescence staining was performed on frozen tissue samples to evaluate the cross-sectional areas (CSAs) of EDL muscle myofibers. Anti-laminin (ab11575, Abcam) was used to outline the myofibers. Images were obtained using an OLYMPUS IX73 microscope (Olympus Co., Japan), and CSA was calculated with ImageJ software (Fiji) with at least 200 fibers per animal.

### 2.7. Measurements of Muscle Contractile Properties

As previously described, EDL muscle contractile properties were measured ex vivo [22]. Each muscle strip (approximately 1 cm in length) was prepared on ice and rapidly mounted in a tissue chamber containing Krebs-Henseleit solution (Sigma, Saint Louis, MO, USA), which was bubbled with a gas mixture of 95% O_2_–5% CO_2_. Muscle extremities were held in spring clips and attached to an electromagnetic force transducer for the measurements. The maximum tetanic forces and the force-frequency curves were used to evaluate muscle force-generating capacity.

### 2.8. Measurements of Proinflammatory Expressions in Skeletal Muscle

Proinflammatory cytokines including tumor necrosis factor- (TNF-) *α*, interleukin- (IL-) 6, and IL-1*β* were detected using commercial enzyme-linked immunosorbent assay kits (HS Quantikine; R&D Systems, USA) according to the manufacturer's instructions.

### 2.9. Measurements of Mitochondrial ROS Production

Mitochondrial ROS production in skeletal muscle was measured using the Amplex RedTM reagent (Life Technologies, CA, USA) as previously described [12]. The fluorescence was normalized to the weight of the dry tissue of controls. In addition, mitochondrial ROS levels in cells were measured using a MitoSOX Red mitochondrial superoxide indicator as previously described [23].

### 2.10. Oxyblot for the Measurements of Protein Carbonylation

Protein carbonylation was measured to evaluate oxidative stress using an Oxyblot Protein Oxidation Detection Kit (Sigma-Aldrich S7150, USA) following the manufacturer's guidelines. Muscle samples were homogenized, and the total protein concentration was determined by the BCA kit (Boxter, Wuhan, China). After derivatization, the proteins were separated, transferred, and then stained with Ponceas S. The acquired protein bands were exposed and captured under the luminescent imaging system (Tanon-6200, China).

### 2.11. RT-qPCR

The real-time quantitative polymerase chain reaction (RT-qPCR) was performed following a standard protocol. The extracted RNA was reverse transcribed using Revert Aid First Strand cDNA Synthesis kit (Invitrogen, Shanghai, China). The RT-qPCR for IL-6, MuRF-1, Atrogin-1, and PGC-1ɑ were implemented using primers to amplify genes as follows: IL-6: forward: 5′-AGTCCCTGCTCGAATCTTCCT-3′, reverse: 5′-TCCCAAGGCAGAACAGATATACC-3′; MuRF-1: forward: 5′-GTGTGAGGTGCCTACTTGCTC-3′, reverse: 5′-GCTCAGTCTTCTGTCCTTGGA-3′; Atrogin-1: forward: 5′-CAGCTTCGTGAGCGACCTC-3′, reverse: 5′-GGCAGTCGAGAAGTCCAGTC-3′; PGC-1ɑ: forward: 5′-AGTCCCTGCTCGAATCTTCCT-3′, reverse: 5′-TCCCAAGGCAGAACAGATATACC-3′; GAPDH: forward: 5′-AGGTCGGTGTGAACGGATTTG-3′, reverse: 5′-G TGTAGACCATGTAGTTGAGGTCA-3′; mRNA expression levels were quantified using the 2-*ΔΔ*Cq method.

### 2.12. Western–Blot Assay

Equal amounts of proteins were resolved by SDS–PAGE, and the proteins were transferred to Hybond ECL membranes (Amersham, Buckinghamshire, UK). The membranes were incubated with primary antibodies including Atrogin-1, MuRF-1, PGC-1ɑ, and GAPDH (Abcam, Shanghai, China) at 4°C overnight. After washing with TBST, the membranes were probed with secondary antibodies and were visualized using an enhanced chemiluminescence system (Kodak, Rochester, NY, USA). GAPDH was used as a loading control.

### 2.13. Statistical Analysis

Data are expressed as mean ± standard deviation (SD). Comparison of means was performed using one-way analysis of variance (ANOVA). Comparisons between two groups were performed by unpaired Student *t*-test. All statistical analyses were performed using R packages. A two-tailed *P* value less than 0.05 was considered significant.

## 3. Results

### 3.1. Systemic Response to CLP and IL-6 Knockout

**Figure 1:**
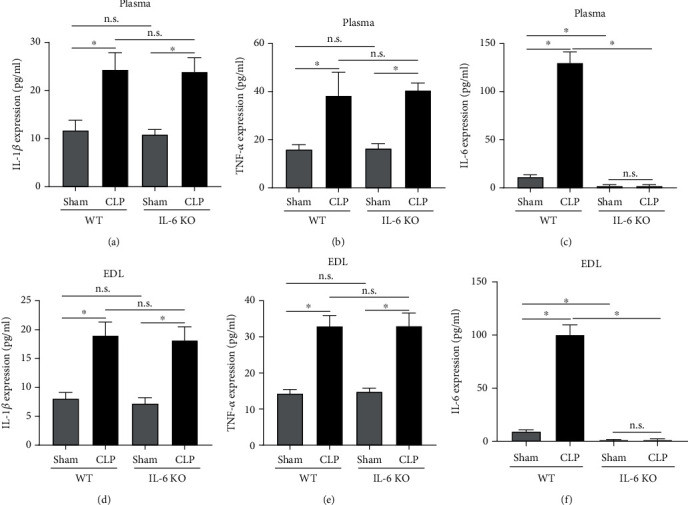
IL-6 KO reduced IL-6 expression in plasma and EDL. (a)–(c) ELISA assays were performed to detect the protein expression of IL-1, TNF-, and IL-6 in plasma of mice; (d)–(f) ELISA assays were performed to detect the protein expression of IL-1, TNF-, and IL-6 in EDL of mice; CLP: cecal ligation and puncture; EDL: extensor digitorium longus. ^∗^*P* < 0.001 (ANOVA followed by unpaired Student *t*-test, *n* = 6).

Compared to the WT-Sham and the IL-6 KO-Sham group, the pH value and the MAP were significantly decreased in the WT-CLP group (all *P* < 0.001). In contrast, the serum levels of lactate were significantly higher in the WT-CLP group than that of the WT-Sham and the IL-6 KO-Sham group (all *P* < 0.001). BP decreased, and observation time increased. At the end of the experiments, the MAP in the CLP group was significantly decreased as compared with the WT-Sham and the IL-6 KO-Sham group (all *P* < 0.001). However, no significant differences in the PaCO_2_, the PaO_2_, and the HCO_3_^−^ were detected between the sham-operated and the CLP groups (all *P* > 0.05). Moreover, IL-6 KO failed to attenuate CLP-induced increases in lactate expression and decreases in MAP of septic mice (all *P* < 0.001). The proinflammatory cytokine expressions were measured by ELISA assays. Our results showed that the protein expression of IL-6 was markedly decreased in the IL-6 KO-CLP compared with the WT-CLP group (*P* < 0.001). However, no significant differences in IL-1*β* and TNF-*α* were documented in skeletal muscles between groups (all *P* > 0.05). Data were summarized in Table 1 and Figure 1.

### 3.2. IL-6 Deficiency Attenuated CLP-Induced Skeletal Muscle Atrophy and Weakness

**Figure 2:**
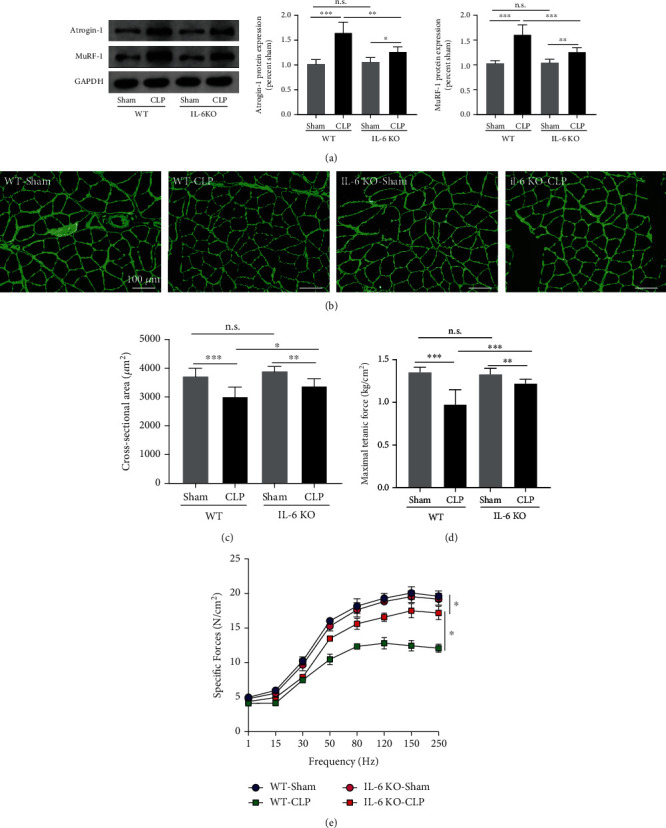
IL-6 KO attenuated CLP-induced skeletal muscle atrophy and weakness. (a) Immunoblots for Atrogin-1 and MuRF-1 in wild-type mice and IL-6 KO mice received sham operation or CLP; immunofluorescence staining (b) was performed to measure the CSA (c) of myofibers (green: anti-laminin was used to outline the myofibers); the maximum tetanic forces (d) and the force-frequency curves (e) were generated to evaluate muscle force-generating capacity. CLP: cecal ligation and puncture; CSA: cross-sectional area. ^∗^*P* < 0.05, ^∗∗^*P* < 0.01, and ^∗∗∗^*P* < 0.001 (ANOVA followed by unpaired Student *t*-test, *n* = 6).

To determine whether CLP induces skeletal muscle atrophy in mice, we evaluated protein degradation, myofiber CSA, and muscle force-generating capacity in septic animals. As seen in Figure 2(a), CLP induces marked increases in the protein expression of muscle atrophy-related genes including Atrogin-1 and MuRF-1 in wild-type mice (all *P* < 0.001). Immunofluorescence staining suggested that the CSA of EDL myofibers was significantly lower in the WT-CLP group than that of the WT-Sham group (all *P* < 0.001) (Figures 2(b) and 2(c)). As depicted by the maximum tetanic forces (Figure 2(d)) and the force-frequency curves (Figure 2(e)), the muscle force-generating capacity of skeletal muscles was markedly downregulated by CLP as compared with the sham operation in wild-type mice (all *P* < 0.01). However, the IL-6 knockout (KO) reversed CLP-induced protein degradation, muscle atrophy, and force loss in mice. Compared to the WT-CLP group, the protein expressions of Atrogin-1 and MuRF-1 were markedly decreased, whereas the CSA of myofibers and the muscle force-generating capacity were significantly increased in the IL-6 KO-CLP group (all *P* < 0.01) (Figures 2(a)–2(e)). Together, these results suggested that we successfully established a murine model of sepsis-associated skeletal muscle atrophy, and the IL-6 KO attenuated CLP-induced skeletal muscle atrophy and subsequent contractile dysfunction.

### 3.3. IL-6 Deficiency Inhibited Mitochondrial ROS Production by Upregulating PGC-1ɑ Expression

**Figure 3:**
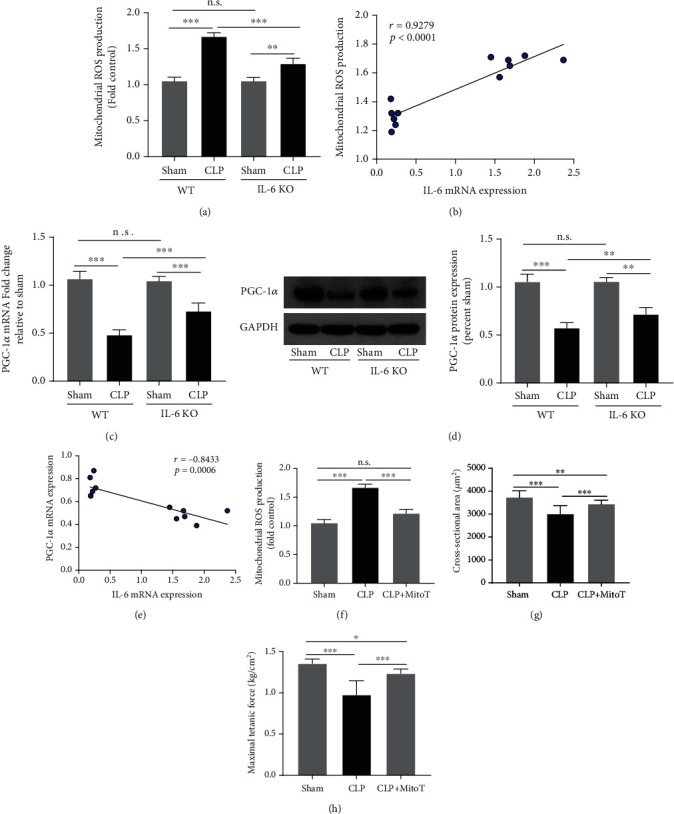
IL-6 KO inhibited mitochondrial ROS production and upregulated PGC-1ɑ expression in septic mice. (a) Mitochondrial ROS production in EDL was measured at the end of experiment; (b) correlation analysis revealed a positive association between IL-6 mRNA expression and mitochondrial ROS production; RT-PCR (c) and western blots (d) were performed to measure the mRNA and protein expressions of PGC-1ɑ in EDL; (e) correlation analysis revealed a negative association between IL-6 mRNA expression and PGC-1ɑ mRNA expression; (f) mitochondria-targeted antioxidant MitoTempol (MitoT) abolished CLP-induced mitochondrial ROS production; (g) MitoTempol treatment increased CSA of myofibers of septic mice; (h) MitoTempol treatment improved muscle force-generating capacity of mice subjected to CLP. CLP: cecal ligation and puncture. ^∗^*P* < 0.05, ^∗∗^*P* < 0.01; ^∗∗∗^*P* < 0.001 (ANOVA followed by unpaired Student *t*-test, *n* = 6).

Here, we observed that the production of mitochondrial ROS in the WT-CLP group was significantly higher than that in the WT-Sham group (*P* < 0.001). In contrast, the mitochondrial ROS production in the IL-6 KO-CLP group was markedly decreased as compared with the WT-CLP group (*P* < 0.01) (Figure 3(a)). Correlation analysis demonstrated a positive association between mitochondrial ROS production and IL-6 expression (*r* = 0.9279, *P* < 0.001). (Figure 3(b)). It has been suggested that PGC-1ɑ is a master regulator of mitochondrial biogenesis and exhibits as a strong antioxidant. In the present study, we found that the mRNA and protein expressions of PGC-1ɑ in skeletal muscles were markedly decreased in the WT-CLP group as compared with the WT-Sham group (all *P* < 0.01) (Figures 3(c) and 3(d)). IL-6 knockout reduced the reduction of PGC1a (all *P* < 0.01). Correlation analysis demonstrated a negative association between PGC-1ɑ mRNA expression and IL-6 mRNA expression (*P* < 0.001) (Figure 3(e)). In addition, the use of mitochondria-targeted antioxidant MitoTempol abolished CLP-induced mitochondrial ROS production in skeletal muscles of septic mice (*P* < 0.001) (Figure 3(f)), which increased the CSA of myofibers (Figure 3(g)) and improved muscle contractile capacity (Figure 3(h)) (all *P* < 0.01). These results suggested that the protective effect of IL-6 KO against skeletal muscle atrophy was probably associated with its antioxidative ability.

**Figure 4:**
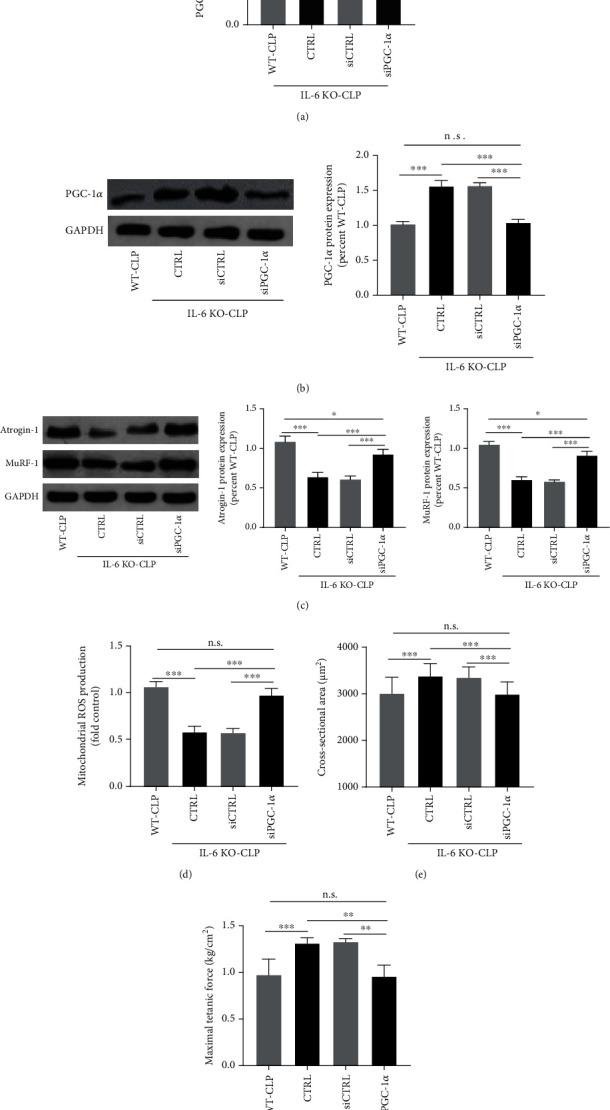
The knockdown of PGC-1ɑ abolished the protective effects of IL-6 KO in CLP-induced skeletal muscle atrophy. RT-PCR (a) and western-blots (b) were performed to determine the mRNA and protein expressions of PGC-1ɑ in skeletal muscle of septic mice after PGC-1ɑ knockdown; (c) the immunoblots for the protein expressions of atrophy-related genes Atrogin-1 and MuRF-1; (d) mitochondrial ROS production was measured after PGC-1ɑ knockdown in septic mice; the cross-sectional area (CSA) of myofibers (e) and the maximum tetanic forces (f) were measured to evaluate muscle atrophy and contractile capacity. CLP: cecal ligation and puncture; CSA: cross-sectional area. ^∗^*P* < 0.05, ^∗∗^*P* < 0.01, and ^∗∗∗^*P* < 0.001 (ANOVA followed by unpaired Student *t*-test, *n* = 6).

To determine whether IL-6 KO inhibited mitochondrial ROS production by upregulating PGC-1ɑ expression, the tail vein injection was conducted to transfect lentivirus-containing shPGC-1ɑ. WB showed a good transfection efficiency (Figure S1). As seen in Figures 4(a) and 4(b), the expressions of PGC-1ɑ in the IL − 6 KO − CLP + shPGC − 1*ɑ* group were significantly lower than that of the IL-6 KO-CLP group (all *P* < 0.01), while no significant differences in PGC-1ɑ expressions were detected between the WT-CLP and the IL − 6 KO − CLP + shPGC − 1*ɑ* group (*P* > 0.05). The immunoblots for the protein expressions of atrophy-related genes Atrogin-1 and MuRF-1 (Figure 4(c)) suggested that the protein degradation was enhanced in the IL − 6 KO − CLP + shPGC − 1*ɑ* group as compared with the IL-6 KO-CLP group (*P* < 0.01). Moreover, the knockdown of PGC-1ɑ largely increased the mitochondrial ROS production in the IL − 6 KO − CLP + shPGC − 1*ɑ* group as compared with the IL-6 KO-CLP group (*P* < 0.01) (Figure 4(d)). There was no significant difference in mitochondrial ROS production between the WT-CLP and the IL − 6 KO − CLP + shPGC − 1*ɑ* group (*P* < 0.05). The measurements of CSA of myofibers and the muscle contractile properties (Figures 4(e) and 4(f)) suggested that the knockdown of PGC-1ɑ abolished the protective effects of IL-6 KO against sepsis-associated skeletal muscle atrophy.

### 3.4. Exogenous IL-6 Induced Mitochondrial ROS-Mediated Proteolysis by Inhibiting PGC-1ɑ Expression Ex Vivo

**Figure 5:**
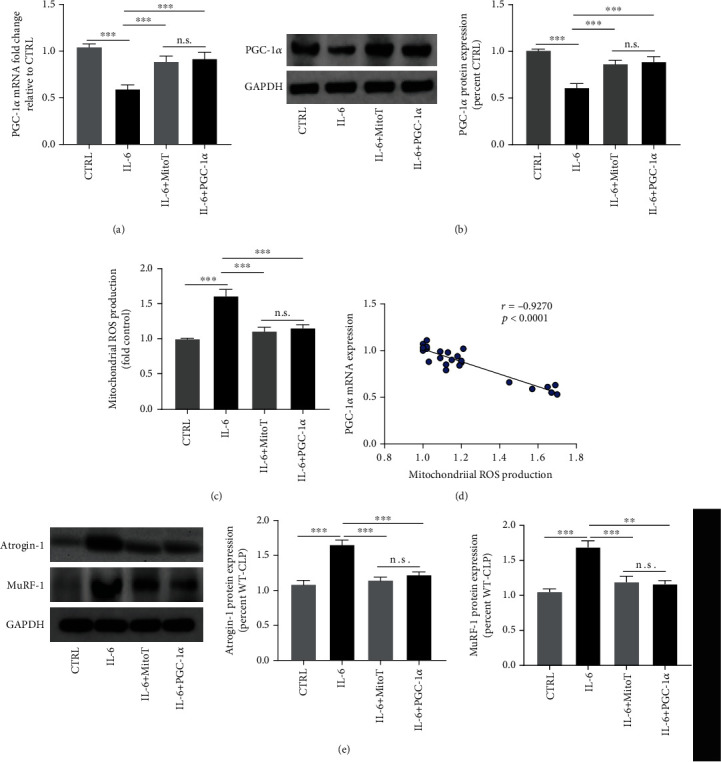
Exogenous IL-6 induced mitochondrial ROS-mediated proteolysis by inhibiting PGC-1ɑ expression ex vivo. RT-PCR (a) and western blots (b) were performed to determine the mRNA and the protein expressions of PGC-1ɑ, respectively; (c) changes in mitochondrial ROS production after IL-6 treatments; (d) correlation analysis revealed a negative associated between PGC-1ɑ expression and mitochondrial ROS production; (e) western blots for the protein expressions of atrophy-related genes Atrogin-1 and MuRF-1 in C2C12 cells after IL-6 treatment; ^∗^*P* < 0.001 (ANOVA followed by unpaired Student *t*-test, *n* = 6).

Our animal experiments suggested that IL-6 deficiency attenuated CLP-induced skeletal muscle atrophy by inhibiting mitochondrial ROS production through the upregulation of PGC-1ɑ. Next, we aim to furtherly elucidate the underlying molecular mechanism by which sepsis promotes protein degradation and skeletal muscle atrophy. C2C12 cells were treated with exogenous IL-6 for 24 hours, and then cells were harvested for analysis. Our results showed that the mRNA and the protein expressions of PGC-1ɑ were decreased after IL-6 treatments (all *P* < 0.01) (Figures 5(a) and 5(b)). In addition, mitochondrial ROS production was markedly upregulated by the treatments of IL-6 in C2C12 cells (*P* < 0.01) (Figure 5(c)). In addition, IL-6 promoted the protein expressions of atrophy-related genes including Atrogin-1 and MuRF-1 (Figure 5(d)). However, both antioxidant MitoTempol and PGC-1ɑ overexpression reversed the IL-6-induced changes in protein degradation. Together, these results suggested that IL-6 promoted protein degradation by inducing mitochondrial ROS production through the downregulation of PGC-1ɑ in C2C12 cells.

## 4. Discussion

The major findings of this study can be summarized as follows: (1) CLP induced apparent skeletal muscle atrophy together with increased IL-6 expression as well as mitochondrial ROS overproduction; (2) the knockout of IL-6 attenuated CLP-induced skeletal muscle atrophy through the downregulation of mitochondrial ROS expression; (3) IL-6 deficiency inhibited CLP-induced mitochondrial ROS production through the upregulation of PGC-1ɑ; together, these results suggested that the IL-6 deficiency attenuated sepsis-associated skeletal muscle atrophy by inhibiting mitochondrial ROS production through the upregulation of PGC-1ɑ.

We established a murine sepsis model by CLP with wild-type and IL-6 KO mice in the present study. Our results suggested that the serum lactate levels were markedly increased, while the MAP was decreased in mice subjected to CLP as compared to sham-operated mice. These data indicated that a murine sepsis and septic shock model had been successfully established. Skeletal muscle atrophy is a long-term complication of sepsis, which results in limited physical activity and poor life quality [24]. The significant physiologic change in response to sepsis is a reduction in muscle force-generating capacity. But in more prolonged models of sepsis, the continued loss of myofiber protein and muscle atrophy are the prominent features [25]. Here in our murine model of sepsis, our results suggested that two major E3 ligases Atrogin-1 and MuRF-1 were markedly upregulated in the EDL of septic mice. In addition, the CSA of myofibers and the muscle force-generating capacity were both decreased by CLP in wild-type mice. These results indicated that a murine model of sepsis-associated skeletal muscle atrophy was successfully established. Laboratory experiments have reported that IL-6 induces skeletal muscle atrophy [26], and the inhibition of IL-6 can attenuate skeletal muscle atrophy caused by denervation [27], cancer cachexia [28], and disuse [29]. The present study first found that IL-6 deficiency and the inhibition of IL-6 trans-signaling alleviated some sepsis-associated muscle atrophy and weakness.

The protective effects of IL-6 KO or IL-6 trans-signaling inhibition appear to be associated with the downregulation of mitochondrial ROS production. Several studies reported that sepsis induces mitochondrial dysfunction and increases the production of mitochondrial ROS in skeletal muscle [30]. It has been widely suggested that mitochondrial ROS is a key regulator of skeletal mass by breaking the balance between protein degradation and protein synthesis [31]. ROS or oxidative stress induces activation of proteolysis-related signalings including UPS, autophagy, and calpains and inhibits the activation of Akt/mTOR signaling that contributes to protein synthesis [32, 33]. It has been reported that IL-6 could induce mitochondrial dysfunction in adipocytes [34] and regulate skeletal muscle mitochondrial remodeling in cancer cachexia [35]. In this study, we found that IL-6 KO reduced CLP-induced mitochondrial ROS production together with decreased expression of muscle atrophy-related proteins. In addition, our in vitro experiments suggested that exogenous IL-6 promoted mitochondrial ROS production and induced protein degradation in C2C12 cells. These results indicated that IL-6 contributes to sepsis-associated skeletal muscle atrophy probably through the upregulation of mitochondrial ROS production and subsequent activation of proteolysis-related signalings.

Some evidence suggested that peroxisome proliferator-activated receptor *γ* coactivator–1alpha (PGC–1*α*), a key regulator of ROS production [36], can protect skeletal muscle against atrophy [37], and the downregulation of PGC–1*α* contributed to the production of ROS and oxidative stress during skeletal disuse atrophy [38]. Here, we observed that sepsis inhibits PGC–1*α* expressions in skeletal muscles, whereas IL-6 KO reversed the expression levels of PGC–1*α*. It has been reported that IL-6/sIL-6R axis activation is critical for the downregulation of renal PGC-1*α* in septic mice [39]. Since PGC–1*α* is a master regulator of mitochondrial biogenesis and can upregulate various antioxidants' expression, it is reasonable to speculate that IL-6 KO reduced CLP-induced mitochondrial ROS production by upregulating PGC–1*α* expression in skeletal muscles. Our result found that the knockdown of PGC–1*α* by siRNA abolished the protective effects if IL-6 KO in CLP-induced skeletal muscle atrophy. In addition, PGC–1*α* overexpression markedly minimized the impact of exogenous IL-6 on mitochondrial ROS production and protein degradation in C2C12 cells. Together, these results first suggested that IL-6 induced mitochondrial ROS production by inhibiting PGC–1*α* expression in skeletal muscle during sepsis.

## 5. Conclusion

In conclusion, our results suggested that IL-6 deficiency protects septic mice against skeletal muscle atrophy by inhibiting mitochondrial ROS production through the upregulation of PGC–1*α* expression.

## Figures and Tables

**Table 1:** Systemic response to CLP and IL-6 knockout.

	WT-Sham	IL-6 KO-Sham	WT-CLP	IL-6 KO-CLP
HR (/min)	323.83 ± 39.38	339.50 ± 35.86	720.17 ± 27.36^∗^	738.17 ± 58.25^∗^
RR (/min)	126.50 ± 5.43	126.83 ± 5.56	264.50 ± 5.68^∗^	251.50 ± 8.87^∗^
pH	7.40 ± 0.04	7.39 ± 0.03^#^	7.27 ± 0.06	7.30 ± 0.03^∗^
PaCO_2_ (mmHg)	40 ± 4	39 ± 5	38 ± 4	40 ± 5
PaO_2_ (mmHg)	102 ± 11	107 ± 8	110 ± 12	104 ± 13
HCO_3_^–^ (mM)	23.7 ± 3.5	27.7 ± 4.8	16.9 ± 3.9	17.8 ± 2.7
Lactate (mM)	1.10 ± 0.13	1.12 ± 0.20^#^	2.71 ± 0.36^∗^	2.67 ± 0.33^∗^
MAP (mmHg)	137 ± 9	135 ± 12^#^	115 ± 18^∗^	110 ± 12^∗^

Data are expressed as mean ± SD; ^∗^*P* < 0.001 vs. WT-Sham group; ^#^*P* < 0.001 vs. WT-CLP group (ANOVA followed by unpaired Student *t*-test, *n* = 6). CLP: cecal ligation and puncture; MAP: mean arterial blood pressure.

## Data Availability

Data are available from the corresponding author on reasonable request.

## Supplementary Materials

Supplementary Materials Figure S1: WB was conducted to verify transfection efficiency, shRNA2 showed a good knockdown effect, and it was taken as the shPGC-1*α* in the study.
