# Supplementary figures and images for: IL-6 Deficiency Attenuates Skeletal Muscle Atrophy by Inhibiting Mitochondrial ROS Production through the Upregulation of PGC-1α in Septic Mice
Source: Oxid Med Cell Longev. 2022 Apr 27;2022:9148246. doi: 10.1155/2022/9148246 (PMC9068301; doi:10.1155/2022/9148246)

Con

shNC

shRNA1

shRNA2

shRNA3

PGC-1 $\alpha$

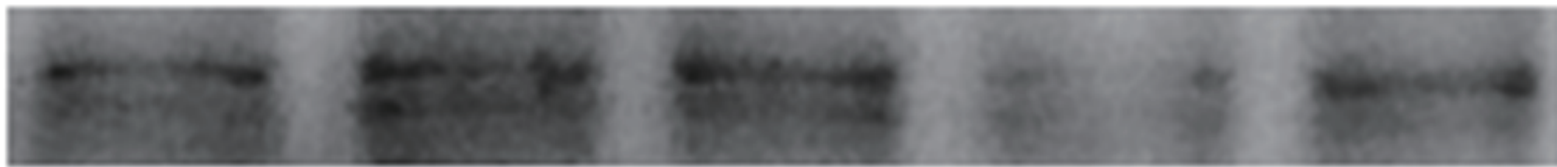

$\alpha$ -tubulin

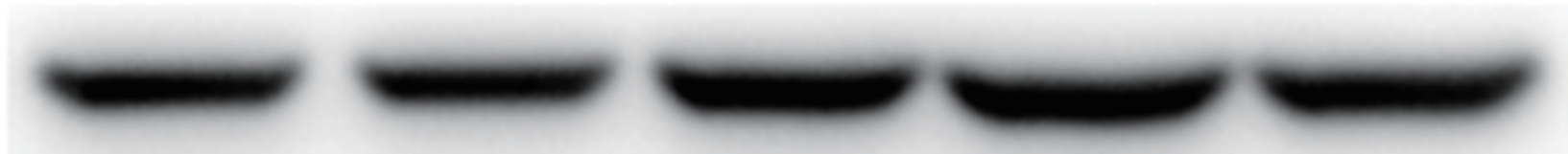

Supplement: Supplementary Materials — Figure S1: WB was conducted to verify transfection efficiency, shRNA2 showed a good knockdown effect, and it was taken as the shPGC-1α in the study. [file 9148246.f1.pdf]
